# Inadequate Completion of Computed Tomography Request Forms of Patients Visiting the Department of Radiology and Imaging in a Tertiary Care Centre

**DOI:** 10.31729/jnma.8327

**Published:** 2023-11-30

**Authors:** Ramswarth Sah, Benu Lohani, Yogendra Prasad Singh

**Affiliations:** 1 Department of Radiology, Tribhuvan University Teaching Hospital, Institute of Medicine, Maharajgunj, Kathmandu, Nepal; 2 Department of Surgery, Tribhuvan University Teaching Hospital, Institute of Medicine, Maharajgunj, Kathmandu, Nepal

**Keywords:** *audit*, *computed tomography*, *form*

## Abstract

**Introduction:**

From both medical and legal points of view, it is vital that computed tomography request forms should be adequately filled up. It is the responsibility of physicians to collect adequate clinical information that justifies the computed tomography examination and the ethical responsibility of radiological technologists and radiologists is to perform only the justified radiological examinations. Thus, a properly filled request form is crucial for understanding the clinical problem, using the proper protocol for avoiding unnecessary radiation exposure and providing concise radiological reports. The aim of this study was to find out the prevalence of inadequate completion of computed tomography request forms of patients visiting the Department of Radiology of a tertiary care centre.

**Methods:**

This descriptive study was performed in the Department of Radiology from 22 April 2021 to 21 April 2022 after receiving ethical approval from the Institutional Review Committee. Computed tomography request forms from emergency, ward and outpatient Departments were used in the study whereas that from other hospitals and clinics were excluded. A convience sampling method was used. The point estimate was calculated at a 95% Confidence Interval.

**Results:**

Out of 470 computed tomography examination forms, the prevalence of inadequate computed tomography request forms was 195 (41.49%) (37.03-45.94, 95% Confidence Interval).

**Conclusions:**

The prevalence of the inadequacy of completion of computed tomography examination forms was higher than other similar studies done in similar settings.

## INTRODUCTION

An adequately filled computed tomography (CT) form, is essential to understand the clinical problem, to use proper protocol for avoiding unnecessary radiation exposure, to provide concise reports with appropriate diagnoses which would prevent any further delay in patient management.^1,2^

CT is used for evaluating injuries, diagnosing symptomatic and asymptomatic patients, and characterizing and staging lesions.^3,4^ Now, many fluoroscopic and conventional radiological procedures have been completely replaced by CT and it accounts for about 50% of medical exposure (except radiotherapy) to ionizing radiation.^5^ International Commission on Radiological Protection has recognized justification as the foundation of radiation protection in the practice of medicine and also suggests the need for effective communication among all concerned and audit of CT examination forms to achieve justification and optimization.^6^

The aim of this study was to find out the prevalence of inadequate completion of computed tomography request forms of patients visiting the Department of Radiology of a tertiary care centre.

## METHODS

A descriptive cross-sectional study was conducted among CT request forms of patients visiting the Department of Radiology, Tribhuvan University Teaching Hospital, Maharajgunj, Kathmandu, Nepal from 22 April 2021 to 21 April 2022. Data were collected from 13 May 2021 to 12 July 2021 after ethical approval from Institutional Review Committee (Reference number: 422 (6-11) E2077-078). All the CT forms of patients from the different Departments during the study period were included. Request forms from other hopital or institutions were excluded. Convenience sampling method was used. The sample size of the study was calculated using the formula:


$$\begin{array}{ll} \text{n} & ={\text{Z}}^{2}\times \frac{\text{p}\times \text{q}}{{\text{e}}^{\text{2}}}\\ & =1.96^{2}\times \frac{0.27\times 0.73}{0.05^{2}}\\ & =303 \end{array}$$

Where,

n = minimum required sample size
Z = 1.96 at 95% Confidence Interval (CI)
p = prevalence of inadequate CT request forms, 27%^1^
q = 1-p
e = margin of error, 5%

The calculated sample size was 303. However, 470 samples were included in the study.

A Self-structured checklist was used as a data collection tool during the study. The data collection technique was the observation of CT request forms. Among all the CT examination request forms received for CT examination of any type and any body part from emergency, any ward and outpatient department (OPD) of TUTH from 9:00 am to 4:00 pm (6 working days per week, Sunday to Friday). The CT examination request forms were systematically observed for the presence of background clinical information, provision of a question to be answered or provisional diagnosis or deferential diagnosis and use of abbreviations. Forms with both backgrounds of clinical information and questions to be answered or differential diagnosis or provisional diagnosis were categorized as adequate while lacking any one of them was inadequate.^1^ All the inadequate CT request forms were also observed for legibility of handwriting and abbreviations present on them were also listed. The handwriting was categorized as legible and illegible with the help of two CT technologists. Handwriting legibility was considered when all words were clear and readable. The handwriting was considered illegible when one or more words were unclear or impossible to identify. Abbreviations used were listed as inappropriate with the help of two radiologists when they did not understand what the abbreviation meant.^1^ On the checklist, all the information (variables) was collected and recorded.

Data were entered and analysis was performed using IBM SPSS Statistics version 16.0. The point estimate was calculated at a 95% Confidence Interval.

## RESULTS

Out of 470 CT examination request forms, the prevalence of inadequacy of the CT request form was 195 (41.49%) (37.03-45.94, 95% CI). Among inadequately filled forms, there were 20 (10.26%) forms that had no background clinical information and 70 (35.90%) had a lack of questions to be answered or provisional diagnosis or differential diagnosis. Forms lacking both the clinical information were 105 (53.85%) (Table 1).

**Table 1:** Inadequate CT examination request forms by type of examinations (n= 195).

CT examination	Inadequate n (%)	Inadequate due to lack of clinical information (A) n (%)	Inadequate due to lack of questions to be answered (B) n (%)	Inadequate due to lack of both A and B n (%)
CT head	38 (19.49)	3 (7.89)	5 (13.16)	30 (78.95)
CT neck	4 (2.05)	1 (25)	1 (25)	2 (50)
CT PNS	5 (2.56)	1 (20)	1 (20)	3 (60)
CT chest	42 (21.54)	4 (9.52)	13 (30.95)	25 (59.52)
CT abdomen	60 (30.77)	5 (8.33)	30 (50)	25 (41.67)
CT temporal bone	6 (3.08)	-	4 (66.67)	2 (33.33)
CT spine	2 (1.03)	-	1 (50)	1 (50)
CT extremities	2 (1.03)	-	1 (50)	1 (50)
CT angiogram	35 (17.95)	6 (17.14)	14 (40)	15 (42.86)
CT guided biopsy	1 (0.51)	-	-	1 (100)
Total		20 (10.26)	70 (35.90)	105 (53.85)

Among all the inadequate CT examination request forms, there were 41 types of abbreviations over 120 (61.54%) request forms. Of the 41 types of abbreviations, 13 (31.71%) were considered inappropriate for not conveying clearly what was meant, which led to a detrimental effect in interpreting the request. Among all the inadequate CT examination request forms, illegible handwriting was found on 19 (9.74%).

## DISCUSSION

The prevalence of inadequacy of filling out the CT examination request form in this study was 195 (41.49%) which was found higher in the study done in a similar setting where it was found to be 27.00%.^1^ In this study, the absence of background clinical information was found on 20 (10.26%) CT examination request forms which was found higher (81.80%) in the study done in a similar setting.^7^ Similarly, a differential or question to be answered or provisional diagnosis was not offered in 70 (35.89%) of the request forms in this study while it was found slightly lower (28.45%) in a similar study.^8^ In a similar study conducted in the United Kingdom, clinical information in terms of brief history and relevant clinical examination was deficient in all request forms.^9^

Regarding abbreviation, there was the use of 41 different abbreviations on 120 (61.53%) out of 195 inadequately filled CT examination request forms in the present study and the most commonly used abbreviation was Ca (ten times) followed by SOB (eight times) further followed by HTN and LOC (six times each). Among 41 abbreviations, ^10^ (31.71%) were considered inappropriate for not conveying clearly what was meant, which led to a detrimental effect in interpreting the request. In this regard, this study showed a high use of inappropriate abbreviations in comparison to a similar international study in which it is 3% only.^1^ Similarly, another study also found the use of non-standardized abbreviations in 6.5% of request forms.^2^ This result is also very low in comparison to the result of the current study in the context of abbreviations.

A study on "the use of abbreviations in medical records in a multidisciplinary world - an imminent disaster" found great variability in the understanding of abbreviations by different groups of health care professionals and recommended that the abbreviations have no place in the multidisciplinary world and their continued use will only lead to eventual clinical error, although abbreviations save time, the observed intergroup variation in the correct interpretation of the abbreviations is unacceptable.^10^

Regarding illegibility of handwriting, 19 (9.74%) out of 195 CT forms were found with illegible handwriting while on the remaining 176 (90.26%) forms, the handwriting was legible. A similar finding was seen in a clinical audit of 444 radiology request forms (illegible handwriting in 8.6%).^2^ Similarly, an audit of 580 radiology request forms in one of the tertiary hospitals of Nigeria found illegibility of the clinician's handwriting on 7.37% of the forms which is slightly lesser than the result (9.74%) of the present study in this regard.^8^

This study has certain limitations. With only 470 samples taken across a variety of CT scan requests, it is difficult to build up a clear picture of patterns within requests. A key point to consider in forming a follow-up audit is the criterion for background clinical information. In this study, any background information was considered adequate despite the amount of detail provided. Across various types of scans, it is difficult to standardize what constitutes adequate clinical information.

## CONCLUSIONS

The prevalence of the inadequacy of completion of computed tomography examination forms was higher than in other similar international studies.
